# Living Beyond the Other

**DOI:** 10.1007/s11089-012-0466-8

**Published:** 2012-07-05

**Authors:** Susana Lauraine McCune

**Affiliations:** Antioch University Seattle, 2326 6th Avenue, Seattle, WA 98121 USA

**Keywords:** Heidegger, Levinas, Death, Psychology, Hospice, Personal narrative

## Abstract

This article attempts to bring philosophy to clinical psychological practice by applying philosophical concepts to autobiographical experience. Through reflective engagement with personal narratives, the author tells three personal stories to illustrate ways in which the concept of Dasein in the philosophy of Martin Heidegger and Emmanuel Levinas’s development of an ethical responsibility to the Other, in tandem with thanatology, helped the author come to terms with existential dilemmas evoked by the deaths of others.

## Introduction

We live in relationship with the Other.^1^ Yet, how do we live when the Other dies?

While providing psychological care with hospice for individuals who were facing their own impending death and for those who were grieving the death of a loved one, I worked within the bio-psycho-social-spiritual model of care that was used at the time by the hospice interdisciplinary team and is still a predominant model among hospice providers (Ferrell and Coyle 2010; Sulmasy 2002). However, this model did not provide answers to the larger, existential questions I faced about the meaning and purpose of living and dying. I could not make sense of the role death played in the lives of my patients and their loved ones or, for that matter, in my own life. Furthermore, I lacked a way to make sense of the pain and suffering of those for whom I was providing care, as well as the suffering I experienced companioning them in their suffering.^2^

Although I felt passionate about the need to provide and improve end-of-life care, I nevertheless experienced sadness and depression. Over time, I became despondent; I felt overwhelmed and empty. This manifested within me as a desire to distance myself from providing more hospice care. As a provider of care, the desire to distance myself from providing care became yet another professional dilemma that further intensified my initial despondence.

In my search for relief from this professional and personal emotional pain, I looked for a more meaningful and effective theoretical orientation, which led me to pursue thanatology.^3^ I also studied philosophy. In doing so, I observed that insights from philosophy are largely missing from thanatological literature. I discovered that, in both philosophical and thanatological literature, the impact of death is discussed in two seminal ways—the death of the self, and the death of the Other.

## Philosophical framework

Thinking about the death of the Other led me to consider writings by the philosopher Emmanuel Levinas. I contemplated Levinas’s poetic development of an infinite ethical responsibility to the Other, which is developed throughout his works. This article will draw predominantly on his ideas outlined in *Is it Righteous to Be? Interviews with Emmanuel Levinas* (2001), *Time and the Other* (1947/1987), and *Emmanuel Levinas: Basic Philosophical Writings* (1996).

In addition to reflecting on the death of the Other, I reflected on my own death. In thinking about my own death, circularity emerged. In order to understand death, I needed to understand its opposite—living or being. In reflecting on being, I needed to understand being’s opposite—not being or the ceasing of being, that is, death. This drew my attention to the writings of German philosopher Martin Heidegger and to the German word *Dasein*, which is a seminal concept in Heidegger’s work. This article draws on ideas in Heidegger’s philosophy as formulated in *Being and Time* (1927/2008; 1927/2010), *Introduction to Metaphysics* (1953/2000), and *Zollikon Seminars: Protocols-Conversations-Letters* (1987/2001)*.*

I acknowledge that there is a historical incommensurability between Heidegger and Levinas. Accounting for these differences, I have attempted to embrace both philosophers’ writings in a non-dualistic, confocal manner. Paradoxically, this approach—examining their work in tandem and seeking synthesis—has revealed much-needed meaning that provided solace from my existential suffering.

## Levinas’s ethics

One idea that emerges again and again in Levinas’s philosophy is his call to an infinite ethical responsibility to the Other. Levinas (1996) declared, “The I before the Other (*Autrui*) is infinitely responsible” (p. 55). Applying this overarching theme to my personal and professional life revealed previously unseen, yet essential, insights about being in relationship with the Other.

Levinas (2001a, b) elaborated in a later interview, “The responsibility for the other is the originary place of identification” (p. 110). This pivotal idea helped me locate myself in relationship with the Other, but more importantly it helped me recognize the essential significance of being *for* the Other. I realized that being in relationship with the Other, and being for the Other, are foundational to one’s identity.

Levinas (2001a, b) distilled another fundamental premise: “It is not a matter of asking ourselves in the name of some abstract law whether or not we ought to give up our lives, but finding reasons for being, for meriting being” (p. 128). I applied Levinas’s assertion—that one is responsible for finding one’s own reasons for being, for meriting one’s being—to the dilemmas that haunted me as I watched suffering and endured the deaths of others. Levinas’s formulation brought forth the insight that it was my responsibility to find a reason for meriting my being. At this point, I recognized that by being with, and by being for, the Other—during their life, at their death, and through their bereavement—I attained merit for my being.

The combination of Levinas’s and Heidegger’s philosophies helped me understand that my relationship with the Other, and my ethical responsibility to the Other, are essential in meriting—or finding value in—my Being.

## Heidegger’s ontology

Concurrent with my discovery of Levinas’s call to an ethical responsibility to the Other, I found that Heidegger’s ontology gave me language to discuss the ineffable, language that is not provided by mainstream psychological theories or the bio-psycho-social-spiritual model.

Heidegger raises anew questions about the truth, the reason, and the meaning of Being (1953/2000; 1987/2001; 1927/2008; 1927/2010). Heidegger’s conceptualizations of Being, not Being, and the givens of life and death gave me a vocabulary with which to articulate previously indescribable concepts related to Being, not Being, the meaning of Being, and the role death plays in Being.

Demske (1970)^4^ articulated the essential role of death in Heidegger’s philosophy: “Death is an ever-present element in the ontological structure of Dasein, a determination of our existence” (p. 25). Demske provided further insight by ascertaining that Heidegger considered death:

a structural determination which serves the function of gathering Dasein into its total existential unity. Death in the existential analysis is not viewed as the “end of the line” for Dasein, but as a possibility-to-be which enables man [sic] both to exist and to understand himself completely. (p. 67)

I came to see, as articulated in Heidegger’s philosophy, that death is an ever-present structural determination, a given of life.

Heidegger’s texts were originally written in German. Translating texts from one language to another is a subtle task fraught with complexity. There is the ever-present risk of oversimplification and mistranslation leading to misunderstanding, as I contend is especially the case for Heidegger’s writings. In the preface to their translation of Heidegger’s most famous work, *Sein und Zeit* (*Being and Time)*, Macquarrie and Robinson (2008)^5^ noted, “Heidegger is constantly using words in ways which are by no means ordinary, and a great part of his merit lies in the freshness and penetration which his very innovations reflect” (p. xxiii).

Dasein is a foundational concept in Heidegger’s lectures and writing. Consequently, understanding his use of the German word is crucial to understanding his approach to philosophy. Scholars have acknowledged the importance of the word Dasein and its related concepts in Heidegger’s ontology (Cohn 2002; Demske 1970; Macquarrie and Robinson 2008; Stambaugh 2010; Inwood 1999; Polt 1999; Richardson 2003; Sheehan 2001). Yet there are inherent problems in translating Dasein from the original German into English.

Dasein and Da-sein have frequently been left untranslated (Macquarrie and Robinson 2008, p. 27; Polt 1999, p. 29). Dasein and Da-sein have also been translated as “being there” (Macquarrie and Robinson 2008, p. 27) and “there-being” (Richardson 2003, p. xliii). Sheehan (2001) offered yet another approach to translating Dasein*:* “I follow Heidegger’s insistence that the *Da* of *Dasein* does not refer to a ‘there’ . . . as well as his suggestions that *Dasein* not be translated as ‘being-here’ or ‘being-there’” (p. 2). Sheehan further clarified in his discussion of Heidegger’s inaugural lecture delivered in 1929 at Freiburg University titled “Was ist Metaphysik?” (“What is Metaphysics”): “The point is to experience *Da-sein,* in the sense that I, the human being, *am* the *Da,* the openness of being for me, insofar as I undertake to preserve this openness, and in preserving it, to unfold it” (p. 3). In his collaboration with Medard Boss, Heidegger (2001) said succinctly: “The basic constitution of human existence may be called *Da-sein*” (p. 4).

In reading texts translated from German into English it may also be helpful to keep in mind that German nouns are capitalized regardless of their position in the sentence; thus, in Heidegger’s writings Dasein is capitalized. Polt (1999) articulated a vital linguistic insight about Heidegger’s use of Dasein: “We should notice that this noun Heidegger uses to designate us is the infinitive form of a *verb*. This suggests that what is distinctive about us is something more like an activity or process than like any sort of *thing*” (pp. 29–30). In his *Introduction to Metaphysics* (1953/2000)*,* Heidegger affirmed his intent that *Dasein* be conceived of as a noun designating a verb, naming an action or process, not a noun naming an object or thing. Heidegger wrote, “Hence there is no Being. All ‘is’ becoming” (p. 102).

Consequently, in my study of his works it became apparent that Heidegger intended Dasein as a noun designating a verb. I also came to understand subtleties of meaning that accompany Dasein as such. I realized that, as Heidegger emphasized in his *ontological difference*,^6^ Dasein is not a thing. In this article, I use the word Dasein to signify the human mode of becoming in which one is aware of one’s own Being and also to connote the process of, place where, and time in which the presencing, revelation, and awareness of Being occur.

## Existentials of Dasein

Dasein is faced with givens, with *existentials*. Critchley (2002) defined existentials as “basic *a priori* structures of Dasein” (p. 9). These are conditions within which being enacts Being, similar to Dilthy’s facticity and Husserl’s a priori structures. Cohn (2002) identified Heidegger’s designation of existentials as “those universal aspects of Being which we have frequently met throughout our various explorations. They are rooted in common human experience—experiential universals so to speak, rather than conceptual ones” (p. 81). Heidegger identified death as one such universal human experience or existential of Dasein. Demske (1970) concluded that for Heidegger “Death is an ever-present element in the ontological structure of Dasein, a determination of existence, which Heidegger calls an ‘*existential*’” (p. 25). In my attempt to make meaning of life and death, I identified several conditions or existentials of Dasein in Heidegger’s ontology that helped me conceive of and talk about living and dying. Here I apply several of them, including*, Being-with, not Being* or *nothingness, possibility, temporality, mortality* or *death,* and *authentically turning toward death*.^7^

In addition to studying the philosophies of Levinas and Heidegger, I also realized that my personal experiences with death were co-constitutive of my worldview, and these experiences were therefore a vital part of what I brought to my clinical work. I had examined my personal history with death in my own therapy, in supervision, and in consultation. Yet something unformulated remained (Stern 1997, 2010). I became aware that enduring questions from personally surviving the deaths of loved ones still haunted me. These questions, while previously beneath my radar, had remained ever-present in my clinical work. I recognized that the next critical step in my development as a therapist—and as a person—hinged on better understanding my personal experiences with the deaths of others. In this article, I present what I have learned thus far about Dasein, the primacy of the encounter with the Other, ethical responsibility to the Other, and death that emerged as I synthesized them through philosophy and the lens of reflective engagement with my personal narratives.

## One clinician, three personal stories

Shortly before my fifth birthday I walked into the kitchen of our family home. I saw mom standing at the sink and my youngest brother, Scotty, who was 18 months old, standing beside her. Mom turned on the faucet and drew a glass of water, then held the glass, helping Scotty drink. His small body immediately stiffened. Then he began thrashing wildly with convulsions. Mom directed me to get my other brother and myself into the car.

I sat in the front passenger seat; Scotty lay on my lap. My mother drove 18 miles to the closest town, which had only one traffic light. As she stopped the car at that red light, two blocks from the doctor’s office, I watched Scotty die.

The next day, in a shed behind the house we found a small green-glass vial and tiny white cubes spilled out onto the ground. This plus the autopsy revealed the cause of death: strychnine. Accidental poisoning.

### Lived experience of existentials: being-with, and not being

While I did not have the words at the instant of my brother’s death, I became aware of existentials that Heidegger identified: Being-with, the Other’s Being, and not Being or nothingness, and the connections among them. Heidegger wrote, “Being-with is in every case a characteristic of one’s own Dasein” (1927/2008, p. 157). Of the connection between Being and not Being, Inwood noted, quoting Heidegger, “Being and the Nothing go together not because they are both indeterminate, but ‘because being itself is in essence finite and reveals itself only in the transcendence of Dasein held out into the Nothing’” (1999, p. 145). Levinas (1947/1987) further helped elucidate my experience of the nothingness that accompanies the death of the Other: “There is, at the same time as the call to an impossible nothingness, the proximity of death” (p. 69).

At the instant my brother died, as a 5-year-old I had touched death and death had touched me. In that instant I learned: My brother’s body was not his Being; I was in relationship with my brother—the Other—one minute, and I was in a relation with not Being or nothingness in the next moment. The death of a being—which left a missing being—paradoxically helped reveal to me the meaning of Being.

The death of my brother also brought about an ungraspable emptiness or nothingess—which I term the *missingness*—that stayed with the living left behind, in a world where the missing Other’s Being had ceased to be. Levinas (1947/1987) captured this deep experience of nothingness and my inability to grasp what had happened in his suggestion that “death is ungraspable” (p. 72). I had just watched my brother die, yet I could not grasp his absence, nor could I grasp the nothingness left behind when he, the Other*,* ceased to be while I continued to be.

### Possibilities lost by turning away: ethical responsibility unfulfilled

Later, death and I met again. When I was an adolescent, my stepfather—the only father I had known—was diagnosed with liver and colon cancer. I cared for him during the 2 years of his illness until his death. He died when I was 15. There was no home-based care, no morphine in the house, no hospice. The prevailing psychological theory of grief remained that of Sigmund Freud’s *decathexis*, as outlined in “Mourning and Melancholia” (1925/1955)*.* This theory, that the vital task of mourning is detachment or decathexis from the deceased, dominated grief theory for nearly 75 years and has determined how everyone is “supposed” to grieve the death of a loved one.

A few years after my stepfather’s death, I came across Elizabeth Kübler-Ross’s book *On Death and Dying* (1969). Kübler-Ross advanced grief theory through a non-sequential, stage conceptualization of response to loss. She also advocated open discussion between the dying person and their loved ones to help complete unfinished life work. Kübler-Ross gave people permission to talk with those who were dying about their impending death. Upon reading the book, I wept both tears of anger and tears of regret.

While reading *On Death and Dying*, I remembered a conversation my stepfather and I had in the spring, about six months before he died. We were standing in the front yard. He said, “I guess I don’t need to plant a garden this year.”

I was confused. He had grown up on a farm and not a year went by without a garden. I asked, “Why not?”

“Because—I won’t be here to harvest it,” he said.

Despite my unformulated knowing of what he was really saying, again I asked, “Why not?”

“I’ll be dead by then,” he answered.

I responded: “Don’t say that. Don’t talk that way.”

I wept tears of regret and anger when I read *On Death and Dying* because I remembered that day. My family and I had not benefited from Kübler-Ross’s work. In the early 1970s, nobody had given me the knowledge or the permission to talk with a dying Other about their dying.^8^ I was filled with guilt and a sense of failure, filled with the feeling that I had failed the Other*.*

Heidegger spoke of death as one of the possibilities of Dasein. Heidegger (1927/2010) wrote, “As a potentiality of Being, Dasein is unable to bypass the possibility of death” (p. 241). Polt (1999) affirmed that, for Heidegger, “Mortality is an ongoing condition of human beings, not a one-time event; it is a *possibility* [emphasis added] . . . that essentially belongs to us” (pp. 86–87). I came to see that mortality governs and reveals possibilities. I have grieved possibilities lost through my inability to be with the Other as they turned toward their death while I turned away.

### Turning toward, turning away

In my search for clarity, I observed that the German prefix *vor-* is used by Levinas in the word *Vor-sicht* (fore-sight) and by Heidegger in *Vorlaufen zum Tode*. The prefix vor relates to time and space. In addition to *“*anticipation,” vor can also mean “in the presence of,” “preparatory,” and “witness” (Messinger 1993, p. 590). Levinas (2001a) theorized that “*Vor-Sicht* (“fore-sight”) toward the death of the other is the beginning of the recognition of the other” (p. 138). Levinas (1947/1987) also asserted, “The problem does not consist in rescuing an eternity from the jaws of death, but in allowing it to be welcomed” (p. 78). Similarly, Heidegger emphasized the importance of one’s attitude toward death throughout life. One of his philosophical contributions is the concept of *Vorlaufen zum Tode*,^9^ which I interpret as a call to authentically turn toward death in anticipation—to face death.^10^ This is not unlike the Samurai or the Sioux warrior who believes every day is a good day to die.

In my stepfather’s case, I distanced myself from him as he authentically turned toward death. On that day in the yard, the Other was facing death. At that moment he was ready to honestly and courageously talk with me about his impending death. In a turn away from the Other and his dying, I denied the possibilities of the moment. I turned away from the abundant possibilities that would have been available if I could have turned toward death with the Other as he faced his death. By turning away, I abandoned possibilities that could have allowed me to be in a more meaningful relationship with the Other, and I disavowed the opportunity to know myself more deeply. These possibilities were forsaken; they became lost possibilities. Although I know I did the best I could with what I knew at the time—because nobody had shown me a better way—I now grieve my unavailability to the possibilities lost as the Other turned toward his death and I turned away because I did not know how to *not* turn away.^11^

## Temporality

Dasein occurs within temporality. Heidegger wrote, “The primordial ontological ground of the existentiality of Dasein, is *temporality*” (1927/2010, p. 224). Elaborating further on the connection between temporality and Dasein, Heidegger asserted, “Remembering this connection, we must show that *time* is that from which Dasein tacitly understands and interprets something like being at all. Time must be brought to light and genuinely grasped as the horizon of every understanding and interpretation of being” (p. 17).^12^

In my understanding of Heidegger’s ontology, we are thrown out of the past and into the present as we project a future, and as Polt (1999) observed, “The future is finite, because it is bounded by mortality” (p. 96). The Other’s future and my future were to be, once more, bounded by mortality.

Death visited me again in 2003. We had celebrated my mother’s 80th birthday. She had lived for over 25 years with a rare degenerative nerve disease. Her health was declining even further due to congestive heart failure. I was designated as her health care power of attorney with responsibility to enact her advance directives. She was in and out of the emergency room and intensive care repeatedly over the course of three months. During one of these episodes, I stood in the hospital hallway with the doctor. He said to me, “Your mother needs a feeding tube.”

I dug down deep within myself and found the strength to say, “No feeding tube.” He looked puzzled. I explained, “My mother has advance directives. No feeding tube.”

“You’re murdering your mother!” He shouted at me. His face and his words are emblazoned in my memory.

It is difficult to overstate how hard it can be to make end-of-life decisions on behalf of an Other*.* I bring up difficult end-of-life subjects for consideration in hopes that no one will face possibilities lost and have to make end-of-life choices for a loved one without direction and backup documentation. Turning toward the possibilities of death with advance care planning can lift burdens that accompany the unpredictability of temporality that often occur during the end of life, an unpredictability that I experienced caring for my mother.

After that experience in the hallway when the doctor shouted at me, my mother’s health improved again. Somewhat unexpectedly, a few weeks later my mother died. I sat with her body, not in intensive care but in a long-term care facility. My brother and I were with her when she took her last breath. After her death, I helped the nurse disrobe and wash her body, and I tied a toe tag on her toe. Then, I had the unenviable task of looking through the telephone book to find a mortuary—so I could call them to come and get her body—as the nursing staff had explained to me I needed to get her body out of the room because they needed the bed for another patient. Even though I had tried to prepare, I was not prepared for the unpredictability of temporality that accompanied the end of her life.

### Possibilities found by turning toward: ethical responsibility to the Other fulfilled

The infiltration of death into life can not only evoke existential dilemmas but also afford possibilities for life completion, transformation for things left unsaid and undone, and transcendence from the pain and loneliness of grief.

During the last three months of my mother’s life, having had the benefit of Kübler-Ross’s work, I told my mother, “Thank you for being a good mom.” I asked her to forgive me for those times I felt I had been a bad daughter. She asked me to forgive her for those times she felt she had been a bad mother. And we talked about her reluctance to die. By authentically turning toward the inevitability of death with the Other, rather than turning away, the possibilities of forgiveness emerged.

During my mother’s last week of life, my brother and I stood by her bed. As we each held one of her hands, talking through tears, we promised her it was okay for her to die. Within a couple of days after this conversation, she died.

By turning toward death together we were able to share tenderness and confession, hold each other, cry together, embrace our failures, and express our deep love. We grew in our own life meaning and grew in our understanding of the meaning of a life completed. Possibilities of Dasein were revealed through our willingness to live life within awareness of death. By turning toward death with the Other, my mother, brother, and I found abundant possibilities. Turning toward the possibilities of death with the Other unveiled the importance of living and dying in relationship with the Other. I realized that death reveals self-understanding through relationship with the Other and, in doing so, death brings reflections of meaning to life. Perhaps the conversation with my stepfather that day in the yard, together with permission to talk with the dying about their impending death which I garnered from the work of Kübler-Ross, enabled me to turn authentically toward death with my mother and, I believe, allowed me to fulfill my ethical responsibility to the Other.

## Theoretical considerations and final reflections

Integrating philosophy and thanatology with reflection on the deaths of my beloved Others has changed me by revealing new interpretations of my life and has disclosed new meanings about the living, the dying, and the loving that connects us to each other throughout Dasein. Bringing philosophy, psychology, and thanatology together to form a multidisciplinary perspective has much to offer in helping us turn toward lived experiences of end of life, death, and bereavement. If, as Peperzak (1993) observed, Levinas’s philosophy emphasized that “the other’s facing me makes me responsible for them, and this responsibility has no limits” (p. 22), then professional caregivers, in particular, who face the Other on a regular basis, can benefit from expanding their own death awareness in order to better help clients, patients, and their families.

I perceive a need to consider end of life, death, and bereavement as normative events that can occur across the lifespan. I propose formulating and more broadly implementing training programs for care-giving professionals that include death awareness. Such programs are essential because clinicians are frequently untrained in having discussions about death, and as a result they often avoid discussions about death altogether. By recognizing the connection between life and death, by facing the givens of Dasein, and by being with the Other in their living, dying, and grieving, we can further develop theoretical bases for end-of-life care, find much-needed language to help us discuss the ineffable, and make more thoughtful, informed decisions about how to live, how to be ethically responsible to the Other, how to love, and how to die.

Reflecting on these personal stories has revealed to me that through my experiences of the deaths of my beloved Others, I bring to my clinical work understanding, sorrow, regret, emptiness, opinions, and insights. Of these I must be constantly aware and grateful. Going forward I hope that I will be able to better fulfill my ethical responsibility to others—to authentically turn toward death with the Other. Perhaps in doing so, I will be blessed and enriched by even deeper meanings of Dasein, which can only be revealed by turning toward death while living, an act that Heidegger, as Inwood noted, referred to as “the highest and uttermost testimony of beyng” (1999, p. 46).^13^

As an older sister, I carried guilt and a sense of failure because I could not stop the Other’s death. As a stepdaughter, I carried regret for not knowing how to turn toward death; I failed in what Levinas described as an ethical responsibility to the Other. As a daughter, I am also blessed with gratitude for having turned toward death before the Other died—for receiving the gift of what Levinas (2001a, b) identified as a “grounding moment of love” (p. 133), a moment that allowed me to fulfill my ethical responsibility for the Other by authentically turning toward death with the Other.

## Footnotes

1 In his preface to *Time and the Other*, French philosopher Emmanuel Levinas notes, “I have always translated *autrui* as the “Other,” with an uppercase “O,” and *autre* as “other,” with a lowercase “o” (Levinas, 1947/1987, p. 30). It is my interpretation that Levinas discussed “the other” in terms that might be more easily understood as a proper English noun. In order to highlight the significance of otherness and bring forward the importance of the idea that we only come to know ourselves through being in relationship with and being for the other, as well as to lift the word “other” out of its common usage in English, throughout this article I capitalize Other.

2 For an excellent discussion of the dynamics of compassion, companioning, and suffering that can emerge during the psychotherapeutic encounter see Orange (2006, 2010).

3 End of life, death, and bereavement are discussed together under the term ‘thanatology*.*’

4 The author expresses her gratitude to William J. Richardson, S. J. for referring her to the work of James M. Demske, for sharing his deeply meaningful experience translating Heidegger’s works, and for conversing about his personal experiences with both Martin Heidegger and Emmanuel Levinas (personal communication, October 2, 2011, Cambridge, MA, at the “Psychology and the Other” conference).

5 Macquarrie and Robinson translated the seventh edition of *Sein und Zeit*, which was published in 1953.

6 Inwood (1999) observed that Heidegger introduced the ontological difference in 1927 “to mark the distinction between BEING ((*das) Sein*) and beings or entities (*das Seiende*)” (p. 46). Polt (1999) wrote, “Heidegger sometimes refers to the difference between Being and entities as *the ontological difference*” (p. 28). Cohn (2002) noted that Heidegger called “whatever belongs to ‘Being’ as such ‘ontological,’ and whatever describes ‘beings’ as ‘ontic.’ The distinction between the two is the ‘ontological difference’” (p. 80). In distinguishing the *ontic* from the *ontological*, Heidegger used the philosophical term ‘ontic’ to designate descriptions of things and describe qualities of existence, as contrasted with his use of *ontology*, which he used in reference to questions about the truth and meaning of Being or the becoming of Being. This distinction has come to be labeled his ‘ontological difference*,*’ in which he differentiated descriptions of being (ontic) from questions related to the meaning of the existence of Being, or Dasein (ontological).

7 Concepts are often articulated with a variety of words and phrases. I offer the following as a basic starting point for rendering German words and phrases in Heidegger’s writings into English: Being-with (*Mitsein)* and Being-with-another (*Miteinandersein*); not Being (*Nicht Sein),* nullity (*Nichtigkeit),* nothingness (*das Nichts*), the not (*Nichtheit)*; possibility (*Möglichkeit*); temporality (*Temporalität*); death (*Tode*); and authentically turning toward death (*Vorlaufen zum Tode*).

8 My discovery of Elizabeth Kübler-Ross’s work just 4 years after my stepfather’s death has continued to provide important guidance throughout my personal and professional life by giving me permission to embrace possibilities, to talk about death, and to talk with a dying Other about their dying. This permission may have allowed me to fulfill my ethical responsibility to the Other going into the future, a future I will discuss later in this article.

9 Stambaugh (2010), translating the 1953 edition of *Sein und Zeit,* rendered *Vorlaufen zum Tode* as “anticipation of death” (p. 420). Yet in her preface, Stambaugh also explicated, “Anticipation is perhaps too weak. Macquarrie and Robinson’s ‘running forward in thought’ seemed a bit awkward. But it may be the better choice” (p. xxv).

10 Several issues emerge in capturing the exact meaning of phrases Heidegger used to emphasize the importance of approaching death during life, including anticipation, running or moving toward, will, and authenticity. Heidegger used *Vorlaufen zum Tode* (frequently translated as “running toward death” and “anticipation of death”) as well as other phrases, including *Sein zum Tode* (being towards death) and *Wille zum Tode* (will to death), to discuss one’s attitudes toward death. Inwood (1999) elaborated, explaining that *Sein zum Tode* (being towards death) and by analogy *Wille zum Tode* (will to death) cover “any attitude one might have to one’s own death, inauthentic (e.g., denying, forgetting, fearing, dwelling on it, suicide, etc.) as well as authentic” (p. 45).

11 Some readers of previous drafts of this article have sympathetically and protectively encouraged me to reconsider my experience. They may have desired to absolve me of my grief and regret. They may have also hoped that I could forgive myself and be free from my regret. I am grateful for their attempts. However, I have come to recognize, in part due to re-telling my personal narratives in this article, that my ability to face my grief and guilt encompasses a challenging but authentic turn toward death. Facing the reality of the regret that remains with me from surviving the deaths of Others has enriched me by helping me move through subsequent deaths of Others in more honest and meaningful ways. My guilt and regret also continue to serve as valuable constant reminders that help me to more authentically turn toward my own death. Rather than eliminate these feelings, I experience value engendered in conscientiously living with my regret and guilt because they have enabled me to respond differently to death.

12 The concepts of *time* and *temporality*, which are central to Heidegger’s conceptualization of Dasein, are presented in this article as I came to understand them through my lived experience. I recognized that Dasein is temporal and finite and is circumscribed by mortality. This awareness emerged as I made meaning of surviving the deaths of my beloved Others.

13 “‘Beyng’ is an archaic form of ‘being’ in middle English” (M. Inwood, personal communication, May 1, 2012). Inwood noted that Heidegger distinguished ‘Beyng’ as an aspect of the *ontological difference* and as the “structure of being” that lies “beyond every entity” (p. 46).
